# Leaf-Methanolic Extract of *Pseudopanax arboreus* (Araliaceae) (L. F. Phillipson) Reverses Amitriptyline-Induced Sexual Dysfunction in Male Rats

**DOI:** 10.1155/2018/2869727

**Published:** 2018-11-15

**Authors:** Egbe B. Besong, G. Ateufack, Smith B. Babiaka, Albert Kamanyi

**Affiliations:** ^1^Department of Zoology and Animal Physiology, Faculty of Science, University of Buea, Buea, Cameroon; ^2^Animal Physiology and Phytopharmacology Laboratory, Department of Animal Biology, Faculty of Science, University of Dschang, Dschang, Cameroon; ^3^Department of Chemistry, Faculty of Science, University of Buea, Buea, Cameroon

## Abstract

The people of the Bayangi tribe (Manyu Division) of Cameroon have used the leaves of *Pseudopanax arboreus* (Araliaceae) traditionally for decades as an aphrodisiac. In order to scientifically investigate this folk claim, we evaluated the effects of the leaf-aqueous extract of *P. arboreus* on the sexual behavior of normal male rats. The present study was designed to assess the effects of the leaf-methanolic extract of *P. arboreus* on amitriptyline-induced sexual dysfunction in male rats. Sexually impaired male rats were randomly divided into 4 groups of 8 rats each. Group 1 received 10 ml/kg distilled water, while group 2 was given 6 mg/kg Viagra. Groups 3 and 4 received 46.5 and 93 mg/kg of the leaf-methanolic extract, respectively. Female rats were made receptive by ovariectomy and subsequent hormonal treatment. Sexual behavior parameters were monitored on days 1, 7, 14, and 21 by pairing each male to a receptive female. The extract-treated rats registered significant decrease in mount latency (ML) and intromission latency (IL); nonsignificant increase in mount frequency (MF), intromission frequency (IF), and penile licking (PL); significant decrease in postejaculatory interval (PEI); contrasting effects in ejaculation latency (EL); and mean intromission interval (MII). Both doses of the extract also provoked a significant increase in relative weight of testes, but had no significant effect on the plasma hormonal profile. These findings are similar to those obtained with normal male rats and show that the leaf-methanolic extract of *P. arboreus* could constitute a potential solution to male sexual impairment.

## 1. Introduction

The male sexual performance is a phenomenon associated with male sexual response cycle and that comprises 4 functional and synchronized phases including libido or sex desire, erection, ejaculation, and orgasm. Libido refers to the biological need for sex and varies among individuals; erection is the firm and enlarged state of the penis, which enables sexual intercourse and other sexual activities, though it is not essential for all of them; ejaculation represents the act of emission and expulsion of semen during sexual intercourse; and orgasm, also called the climax of sexual intercourse is the moment of most intense pleasure. The male sexual activity is termed normal if all these phases or steps occur timely and sequentially; and any delay or disorder in either of the phases or steps is referred to as male sexual dysfunction or impairment (MSD) [1, 2]. Consequently, disorders of the male sexual response cycle are functionally classified into 4 (four) categories: disorder of sex desire or libido, erectile problems, ejaculation disorder, and orgasm disorder. In other words, a man is sexually impaired if he lacks or loses sexual desire, is unable to have an erection sufficient for pleasurable intercourse, does not ejaculate or experiences delayed ejaculation, ejaculates before he or his partner desires, feels pains during intercourse, and/or has prolonged erection [3]. About 10% of men worldwide suffer from MSD. Data on its prevalence in Africa are scanty, while those on its prevalence in Cameroon are unavailable. Factors responsible for MSD are generally grouped into psychogenic (stress and depression), organic (anatomical causes and androgen deficiencies), environmental (obesity, hyperlipidemia, and type II diabetes), and pharmacological treatments such as chronic administration of antidepressants.

As drugs used to treat depression and other psychological disorders, antidepressants are classified into 3 major groups: (1) tricyclic antidepressants (TCAs) which account for about 30% of MSD and have been reported to be implicated in decreased libido, erectile dysfunction, delayed orgasm, and impaired ejaculation; (2) monoamine oxidase inhibitors (MAOIs), responsible for about 40% of MSD cases and having similar effects to those of the TCAs; and (3) selective serotonin reuptake inhibitors (SSRIs), blamed for between 58% and 73% of MSD cases and having effects similar to those of the first two groups [4]. All antidepressants induce MSD by altering the availability and actions of neurotransmitters or hormones that intervene in the male sexual response cycle either at the central or the peripheral level [5–7]. Statistics indicate a global prevalence of antidepressant-induced MSD (AIMSD) of about 24.9%. Unfortunately, statistics on its prevalence in Africa and Cameroon are not available to the best of our knowledge. The use of antidepressants remains a common practice in most societies today, especially in African and Cameroonian rural settings. Focus on their study has recently increased due to their widespread use and prolonged administration. Some like Valium, Proscar, tranylcypromine, amitriptyline, and their relatives have been extensively used and can be considered “over-the-counter” drugs. Many patients especially in Africa, South of the Sahara, including Cameroon, depend on automedication including the use of antidepressants with little or no knowledge on the dosage or possible side effects. They are therefore potential sufferers of AIMSD. Male sexual impairment, in addition to the heavy psychological toll and loss of self-esteem caused on the individual, remains one of the major causes of male infertility and accounts for about 25% of infertility among couples [8]. In general, sexual disorders ought to be remedied because whether an individual is depressed or not, he needs to procreate (reproduce) to ensure the continuity of his lineage [9–11].

Existing therapies against antidepressant-induced and other forms of MSD include psychotherapy, drug therapy, penile surgery, vacuum constrictive devices/constrictive rings, vibratory stimulation, vascular reconstruction, erectogenic drugs (local vasoactive agents), prosthesis, treating the inciting disease, switching from drugs with higher sexual dysfunction effects to those with less sexual dysfunction effects, adjunctive treatment (drug holiday and dosage reduction), etc. The use of most of these approaches is hindered by a lot of setbacks such as cost, pain, side effects, patient's acceptance, etc. Many patients with MSD respond well to the pharmacological treatments that are currently available, but there are still groups of patients in whom the response is unsatisfactory. Again, many pharmacological agents available today do not target all the phases of the male sexual response cycle. Furthermore, it has been demonstrated that most pharmaceutical or standard sexual impairment drugs are known to provoke undesirable secondary or side effects [12–14]. A main and serious problem therefore arises: drug efficacy and safety cycle. Thus, the searching for new agent targeting at numerous phases of sexual response cycle which is more efficient, cheaper, and safer has gained much concentration as many sufferers of MSD rely on medicinal plants to remedy this pathology.

The people of the Bayangi tribe (Manyu Division) of Cameroon have used the leaves of *Pseudopanax arboreus* (Araliaceae) traditionally for decades as an aphrodisiac. The family Araliaceae is closely related to the family Apiaceae and family Pittosporaceae [15]. Members of these families have been demonstrated to possess aphrodisiac potentials. Other members of the family Araliaceae include herbs such as ginseng *Panax* spp., a native of Korea and used as medicinal herb. Traditionally, the plant is used to treat hypertension and MSD with their respective posologies. In treating hypertension, the sufferer orally ingests a daily volume of about 200 ml of its leaf-maceration for a duration of 3-4 days, whereas its posology in treating MSD comprises oral administration of a glassful (about 250 ml) of its leaf-maceration thrice a day for a period of 21 days. In a previous study, [16], we evaluated the in vivo effects of the leaf-aqueous extract of *P. arboreus* on the sexual behavior of normal male rats. The present experiment was therefore designed to study the effects of the leaf-methanolic extract of *P. arboreus* on amitriptyline-induced sexual dysfunction in male rats.

## 2. Materials and Methods

### 2.1. Chemicals and/or Products

Products used in this study were of analytical quality and included amitriptiline hydrochloride 25 mg (Qualitest Pharmaceuticals Inc, Malvern, PA, USA); penicillin G and sildenafil citrate (Viagra) (Pfizer Inc, USA); estradiol and progesterone (Sigma Chemicals, USA); bioassay kits for FSH (DRG Diagnostics, Germany), LH (DRG Diagnostics, Germany), testosterone (Omega Diagnostics LTD, Scotland, UK); ethyl-ether (Mark and Baker LTD, Dagenham, England), and NaCl which were all purchased and handled under recommended conditions until used.

### 2.2. Plant Material

Fresh leaves of *P. arboreus* were harvested from the tropical rainforest of Mamfe, under the guide of a local tradipractitioner and poacher who confirmed the plant's identity based on its local vernacular name. A full branch and an attached flower of the plant were carefully preserved in a local newspaper and taken to the National Herbarium in Yaounde, for authentication. The fresh leaves were chopped into smaller pieces, air-dried under shade for about two months, and ground using an electric grinder. Three hundred grams of the ground powder were introduced into 3000 ml of methanol, kept for 72 hours accompanied by intermittent mechanical agitation. It was then filtered using the laboratory test sieve (Endecotts Ltd., England) of 38 *µ*m aperture. This was followed by solvent evaporation in a rotavapor under reduced pressure to yield 47.54 g of paste giving a 15.85% yield of extraction. Part of the yield was submitted to the Laboratory for Plant and Organic Chemistry of the Department of Chemistry, University of Buea for phytochemical screening. Meanwhile, administrative doses were determined following the tradipratitioner's directives and screening tests.

### 2.3. Animals

#### 2.3.1. Raising of Animals

Animals used were rats of the Wistar strain of either sex raised in the Animal House of the Zoology and Animal Physiology Department of the Faculty of Science, University of Buea, under standard conditions of temperature, humidity, and a 12-h light/dark cycle. They were fed with a standard laboratory diet, and water was available ad libitum.

#### 2.3.2. Ovariectomy and Induction of Estrus in Females

This was done according to the methodology described in [17] and in our previous study [16]. Briefly, a total of 30 females obtained from the breed were starved for 24 hours and prepared for surgical operation. They were weighed and given an intraperitoneal injection of 0.02 ml/100 g body weight diazepam followed by 0.01 ml/100 g body weight ketamine, as anaesthesia; the two injections were separated by a 5-minute interval. After the onset of anaesthesia, the right and left lumbar dorsa of each female was shaved and the exposed skin prepared for aseptic surgery (97% alcohol wipe). A 1-2 cm dorsal flank incision penetrating the abdominal cavity was made for each ovary and the par-ovarian fatty tissue identified and retracted. The exposed ovary and its associated oviduct were severed after making a ligature at the anterior zone to prevent bleeding. The peritoneum and skin were stitched, followed by an intramuscular injection of 0.2 ml of penicillin G to prevent any postsurgical infection and oral administration of diclofenac capsule at the dose of 30 mg/kg as analgesia.

About 14 days following surgery, each ovariectomised female was given 66.67 *µ*g of estradiol benzoate solution subcutaneously to bring them to estrus. This was followed 48 hours later by another subcutaneous injection of 600 *µ*g progesterone solution. The progesterone was administered 6 hours before pairing each female with a sexually experienced nonexperimental male, and only those exhibiting good sexual receptivity (solicitation behavior and lordosis in response to mounting) and no rejection behavior were employed in the experiment [16, 18].

#### 2.3.3. Training of Males for Sexual Experience

About 300 sexually matured males obtained from the breed were used. Each male was put in a transparent cage and 30 minutes later, a receptive or estrus female was introduced into the cage. The sexual activity of the male including mount, intromission, penile licking, and ejaculation were noted daily for 2 hours. This exercise lasted 7 days, and only those sexually active were used subsequently in the study.

#### 2.3.4. Induction of Male Sexual Dysfunction (MSD)

This was done according to the method described in [11, 19, 20]. Sexually trained males obtained above were divided into two groups: A and B. Group A served as control and was given vehicle only (distilled water), while B served as the drug-treated group and was administered an oral dose of 10 mg/kg body weight of amitriptyline suspension (suspension prepared daily in distilled water). The 10 mg/kg dose of amitriptyline hydrochloride was selected considering the 400 mg maximum human daily dose (HED) from which the animal equivalent dose (AED) was determined by extrapolating it in animals using the methods described in [21]. Treatment was given once a day and lasted for 56 days (8 weeks). At the end of the 8^th^ week (on day 57 of treatment), they were randomly selected and used for assessment of mating behavior parameters, relative weight of sex and accessory organs, and hormonal profile.

#### 2.3.5. Male Sexual Dysfunction (MSD) Screening

*(1) Assessment of Mating Behavior*. The method used in training for sexual experience and in evaluating sexual behavior in other rat models [16, 22, 23] was repeated here. Briefly, each water-treated and amitriptyline-treated male was paired separately to a receptive female, on the last day (day 56) of dosing and 30 minutes after receiving the substance. Observations were conducted in the dark phase (as from 20:00 local time) of the light-dark cycle under dim light and very quiet conditions. Each test session was considered ended when mount latency (ML) and postejaculatory interval (PEI) were 20 minutes. The following performance parameters were assessed:

- Mount: this is when the male rat raised the forelimbs and gripped the female followed by the movement of its pelvic region towards the vagina of the female.
- Intromission: this is the thrusting of the pelvic region of the male rat into pelvic region of the female followed by the penetration of the erect penis into the female's vagina.
- Penile licking: this is when the male bent and licked the penis without mounting or intromission.
- Ejaculation: this is when the male gripped the female with the latter raising its snout in an upward direction. In rats, it often comes after a series of successive mounts and intromissions.

From these parameters, the following indices were determined or calculated:

- Mount latency (ML): this is the time interval from the introduction of the female into the cage until the first mount. This varies between a few seconds to minutes and measures the degree of arousal or sexual interest or libido of the male. It is often expressed or measured in seconds.
- Mount frequency (MF): this is the total number of mounts preceding ejaculation. It is also a measure of the degree of the male's sexual interest or libido.
- Intromission latency: this refers to the time interval from the introduction of the female into the cage until the first intromission. It ranges from a few seconds to minutes and measures the degree of sexual excitement and erection. It is measured in seconds and varies with the individual and the circumstances.
- Intromission frequency (IF): this represents the number of intromissions preceding an ejaculation. Like intromission frequency, it measures the degree of erection.
- Ejaculation latency (EL): it is the time interval from the introduction of the female to the first ejaculation. It ranges from a few seconds to minutes and determines the efficiency of copulation in rats.
- Postejaculatory interval (PEI): it is the time interval between an ejaculation and the next first mount. Generally in rats, it ranges from 6 to 10 minutes. It measures the refractory period that comes after ejaculation.
- Mean intromission interval (MII) or intercopulatory efficiency (ICE): it was calculated as ejaculation latency divided by intromission frequency. It measures both the degree of erection and the efficiency of copulation.

*(2) Sex Organ Relative Weight Assessment*. Males destined for this purpose were randomly selected from either group (distilled water-treated and amitriptyline-treated) and starved for 24 hours. They were sacrificed under ethyl-ether as anaesthesia, and the following organs were isolated: testes, epididymis, vas deferens, prostate, seminal vesicle, and penis. Each was rinsed thoroughly and wiped with clean absorbent paper, carefully freed from all connective tissue and then weighed using an electronic scale (NVT 1601/1, OHAUS Corporation, USA). Their individual weights were then expressed as a percentage of the total body weight.

*(3) Hormonal Profile Assessment*. Alongside the sex organs, blood was collected using a 5 ml syringe through cardiac puncture and immediately transferred into heparinized test tubes. It was kept for 24 hours after which the supernatant was collected and put into test tubes. It was then centrifuged for 15 minutes at 2500 rpm. At the end of this, the supernatant was again collected. Plasma concentrations of FSH, LH, and testosterone were determined using enzymatic kits and standardised reagents and following protocol prescribed by the manufacturer. In each case, a blank solution was prepared to help calibrate or standardise the ELIZA reader [24].

Compared to the control group, animals were considered sexually impaired when they showed minimum (25%) reduction in sexual activity such as low libido or poor sexual arousal (mount and intromission latencies ≥180 seconds); poor sexual vigour and poor sexual performance (mount and intromission frequencies ≤10); very low (≤300 seconds/≤5 minutes) or high (≥900 seconds/about 15 minutes) ejaculation latency; or low ejaculatory frequency (zero ejaculation); penile licking values ≤3.5; and mean intromission values ≥30 seconds. Also considered were low sera hormonal profile of FSH, LH, and testosterone, low relative weight of sex organs, as well as poor sperm characteristics (low percentage motility and total sperm count). This category of animals was then incorporated into the next phase of the study. They were then allowed for 255 hours (11.45 days) (being the wash-out time of amitriptyline) at the end of which they were again randomly selected and assessed for mating behavior, relative weight of sex and accessory organs, and hormonal profile.

### 2.4. Animal Repartition and Treatment

Males confirmed sexually impaired were randomly divided into 4 groups of 8 rats each. Group 1 received 10 ml/kg distilled water and served as the negative control, while group 2 was given 6 mg/kg Viagra to serve as the positive control. Groups 3 and 4 received 46.5 and 93 mg/kg of the methanolic extract, respectively. All substances including distilled water, Viagra, and extracts were administered orally using the metal oropharyngeal cannula.

Treatment lasted for 58 days, and sexual observations were done on days 1, 7, 14, and 21 following the methodology described earlier. Each test session was considered ended when mount latency (ML) and postejaculatory interval (PEI) was 20 minutes. At the end of the 58-day treatment period, the animals were sacrificed under ethyl-ether and the selected organs and serum collected for the relative weight, sperm characteristics, and hormonal assay as described earlier.

### 2.5. Acute Toxicity Test

The acute toxicity study was done following the Organization for Economic Co-operation and Development (OECD) guideline 425 [25]. Before the oral administration of a single dose of the test samples, the rats were deprived of food for 3 hours then divided into 3 groups of 7 rats each. Animals of group 1 were administered distilled water to serve as the control, while those of groups 2 and 3 received 2000 and 5000 mg/kg of the methanolic extract, respectively. All animals were observed for general behavioral changes, as well as symptoms of toxicity and mortality after treatment for the first four (critical) hours, then over a period of 24 hours, thereafter daily for 14 days. Body weights were measured daily while abnormal findings were recorded with the time of onset and disappearance.

### 2.6. Ethical Considerations

The research protocol was approved by the University of Buea Institutional Animal Care and Use Committee (UB-IACUC), and an ethical clearance number (UB-IACUC No. 003/2018) was given.

### 2.7. Statistical Analyses

Values were expressed as mean ± SEM. Mean values were calculated for each animal and quantitative comparison between groups established from those means. One-way analysis of variance (ANOVA) followed by Duncan test using SPSS for windows version 20.0 was done to test for the significant level at *p* < 0.05.

## 3. Results

### 3.1. Results of the Phytochemical Screening of the Leaf-Methanolic Extract of *P. arboreus* (Araliaceae) (L. F. Phillipson)

The phytochemical screening of the leaf-methanolic extract of *P. arboreus* revealed the presence of alkaloids, flavonoids, phenols, steroids, saponins, and triterpenoids.

### 3.2. Effects of the Methanolic Extract of Leaves of *Pseudopanax arboreus* (Araliaceae) (L. F. Phillipson) on the Sexual Behavior of Amitriptyline-Induced Sexually Impaired Male Rats

#### 3.2.1. Effects on Copulatory Parameters

**Figure 1:**
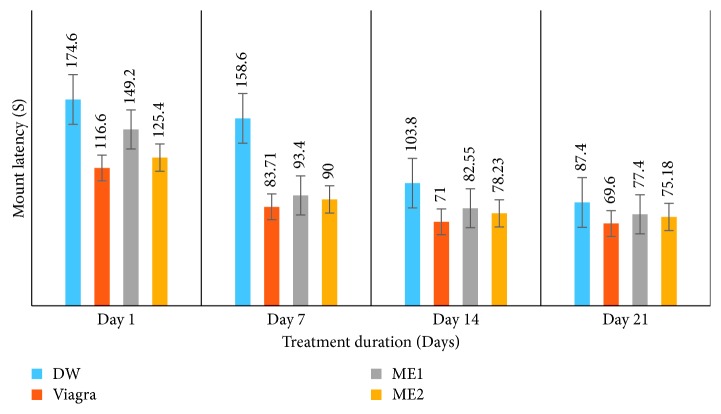
Effects of the leaf-methanolic extract of *P. arboreus* (ME1 and ME2) on the mount latency (ML) (s) of amitriptyline-induced sexually impaired male rats. Values presented as mean ± SEM; DW: distilled water; ME1: methanolic extract dose 1 (46.5 mg/kg); ME2: methanolic extract dose 2 (93 mg/kg).

**Figure 2:**
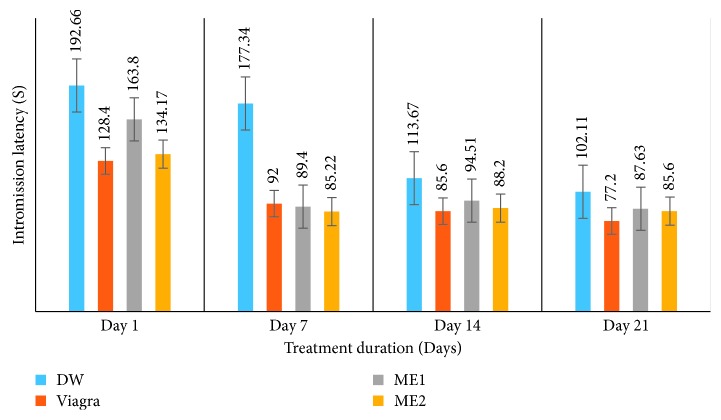
Effects of the leaf-methanolic extract of *P. arboreus* (ME1 and ME2) on the intromission latency (IL) (s) of amitriptyline-induced sexually impaired male rats. Values presented as mean ± SEM; DW: distilled water; ME1: methanolic extract dose 1 (46.5 mg/kg); ME2: methanolic extract dose 2 (93 mg/kg); (s) seconds.

*(1) Effects on Moun Latencyt (ML) (s) and Intromission Latency (IL) (s)*. Subjection of amitriptyline-induced sexually impaired male rats to leaf-methanolic extract at 46.5 and 93 mg/kg (ME1 and ME2, respectively) for a 21-day treatment period resulted in a significant (*p* < 0.05) decrease in mount latency (ML) and intromission latency (IL) in a dose-dependent manner. Compared to the distilled water-treated group, the leaf-methanolic extract-treated sexually impaired rats recorded lower values of ML and IL, though relatively higher than those in rats treated with Viagra (sildenafil citrate) (Table 1 and Figures 1 and 2). ML values dropped from 174.60 ± 16.94 to 87.40 ± 8.62 (DW-treated rats), from 116.60 ± 5.73 to 69.60 ± 16.38 (Viagra-treated rats), from 149.20 ± 9.88 to 77.40 ± 7.30 (ME1), and from 125.40 ± 9.18 to 75.18 ± 13.86 (ME2), whereas IL values dropped from 192.60 ± 16.83 to 102.11 ± 21.11, from 128.40 ± 5.68 to 77.20 ± 17.88, from 163.80 ± 8.20 to 87.63 ± 8.76, and from 134.40 ± 8.56 to 85.60 ± 15.93 in DW-, Viagra-, ME1-, and ME2-treated rats, respectively, from day 1 to day 21 (Table 1 and Figures 1 and 2).

**Figure 3:**
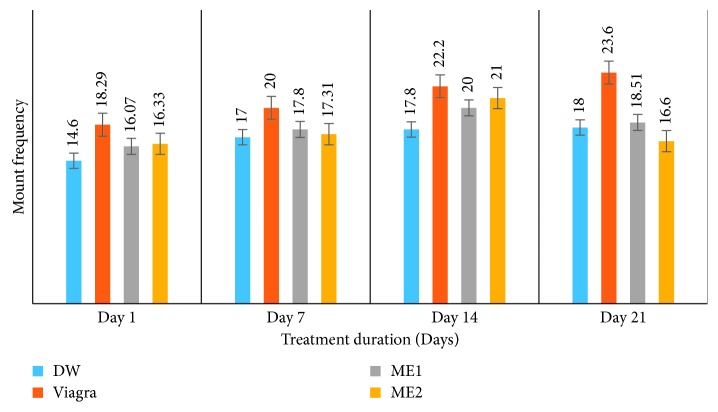
Effects of the leaf-methanolic extract of *P. arboreus* (ME1 and ME2) on the mount frequency (MF) of amitriptyline-induced sexually impaired male rats. Values presented as mean ± SEM; DW: distilled water; ME1: methanolic extract dose 1 (46.5 mg/kg); ME2: methanolic extract dose 2 (93 mg/kg).

**Figure 4:**
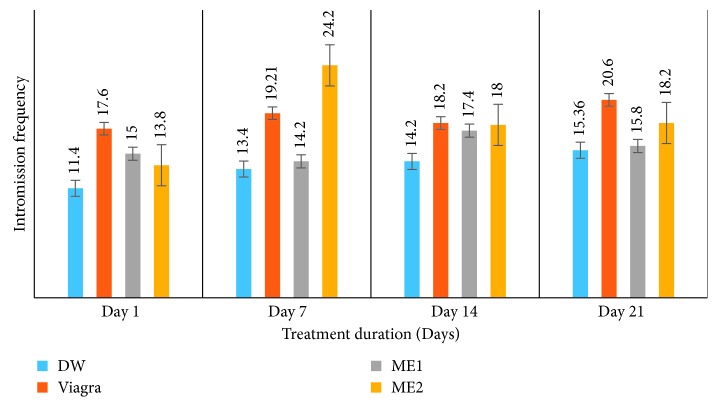
Effects of the leaf-methanolic extract of *P. arboreus* (ME1 and ME2) on the intromission frequency (IF) of amitriptyline-induced sexually impaired male rats. Values presented as mean ± SEM; DW: distilled water; ME1: methanolic extract dose 1 (46.5 mg/kg); ME2: methanolic extract dose 2 (93 mg/kg).

*(2) Effects on Mount Frequency (MF) and Intromission Frequency (IF)*. Like with the ML and IL, treatment of amitriptyline-induced sexually impaired male rats with either dose of the leaf-methanolic extract of *P. arboreus* induced an increase in MF and IF throughout the treatment period, though in a nonsignificant (*p* < 0.05) manner compared to the distilled water-treated animals (Table 2 and Figures 3 and 4).

**Figure 5:**
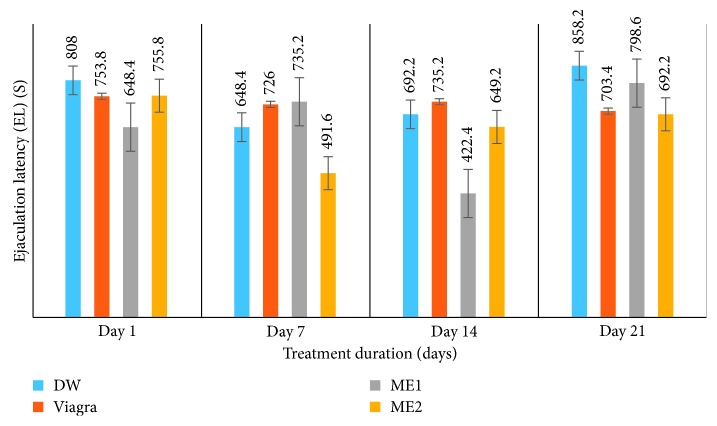
Effects of the leaf-methanolic extract of *P. arboreus* (ME1 and ME2) on the ejaculation latency (EL) (s) of amitriptyline-treated male rats. Values presented as mean ± SEM; DW: distilled water; ME1: methanolic extract dose 1 (46.5 mg/kg); ME2: methanolic extract dose 2 (93 mg/kg).

**Figure 6:**
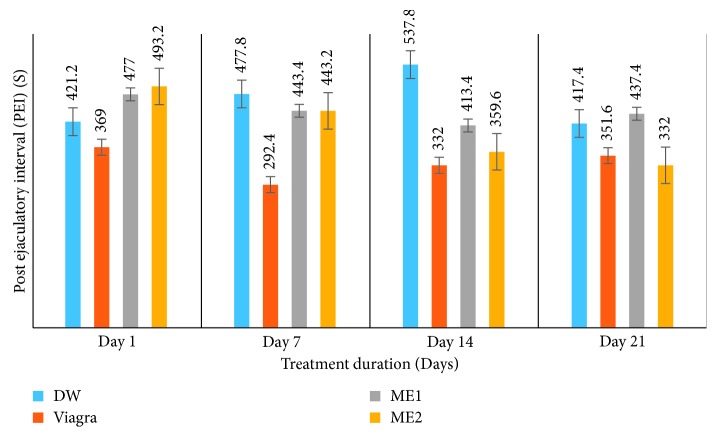
Effects of the leaf-methanolic extract of *P. arboreus* (ME1 and ME2) on the postejaculatory interval (PEI) (s) of amitriptyline-induced sexually impaired male rats. Values presented as mean ± SEM; DW: distilled water; ME1: methanolic extract dose 1 (46.5 mg/kg); ME2: methanolic extract dose 2 (93 mg/kg).

*(3) Effects on Ejaculation Latency (EL) (s) and Postejaculatory Interval (PEI) (s)*. Treatment of amitriptyline-induced sexually impaired male rats with the leaf-methanolic extract of *P. arboreus* at the 46.5 and 93 mg/kg (ME1 and ME2, respectively) doses induced contrasting effects on EL, compared to the distilled water- and Viagra-treated animals.  Table 3 and Figure 5 show a significant (*p* < 0.05) decrease in EL values from day 1 to day 21 in animals treated with ME2, like those of the Viagra-treated rats; whereas rats that received the ME1 dose recorded a significant (*p* < 0.05) increase in EL values from day 1 to day 21, like those of the DW-treated rats. Both doses of the plant extract provoked similar effects in the PEI of rats, compared to the DW and Viagra-treated groups. PEI values in extract-treated rats at either dose significantly (*p* < 0.05) decreased from day 1 to day 21, while a nonsignificant (*p* < 0.05) decrease in PEI values was recorded in both control groups (Figure 6).

**Figure 7:**
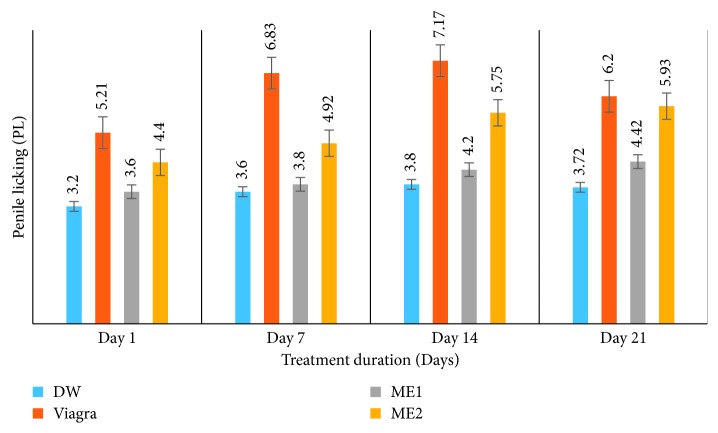
Effects of the leaf-methanolic extract of *P. arboreus* (ME1 and ME2) on the penile licking of amitriptyline-induced sexually impaired male rats. Values presented as mean ± SEM; DW: distilled water; ME1: methanolic extract dose 1 (46.5 mg/kg); ME2: methanolic extract dose 2 (93 mg/kg).

**Figure 8:**
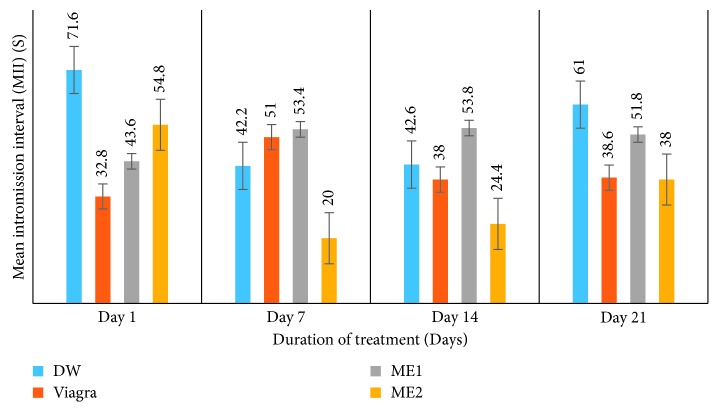
Effects of the leaf-methanolic extract of *P. arboreus* (ME1 and ME2) on the mean intromission interval (MII) (s) or intercopulatory efficiency (ICE) (s) of amitriptyline-induced sexually impaired male rats. Values presented as mean ± SEM; DW: distilled water; ME1: methanolic extract dose 1 (46.5 mg/kg); ME2: methanolic extract dose 2 (93 mg/kg).

*(4) Effects on Penile Licking (PL) and Mean Intromission Interval (MII) (s) or Intercopulatory Efficiency (ICE) (s)*. Administration of the leaf-methanolic extract of *P. arboreus* at either dose induced an increase in PL values from day 1 to day 21 of treatment, although statistically they were not significantly different; in spite of that, greater values were recorded in extract-treated rats than in DW-treated animals (Table 4 and Figure 7). Generally, the effects of the extract on MII were contradictory among the two doses (Table 4 and Figure 8). Animals treated with the ME2 dose showed a nonsignificant (*p* < 0.05) decrease in MII from day 1 to day 21, similar to the distilled water-treated rats, whereas those treated with the ME1 dose recorded a nonsignificant increase in MII values from day 1 to day 21 of treatment, similar to those that received Viagra.

#### 3.2.2. Effects on the Relative Weight of Sex and Accessory Organs

**Figure 9:**
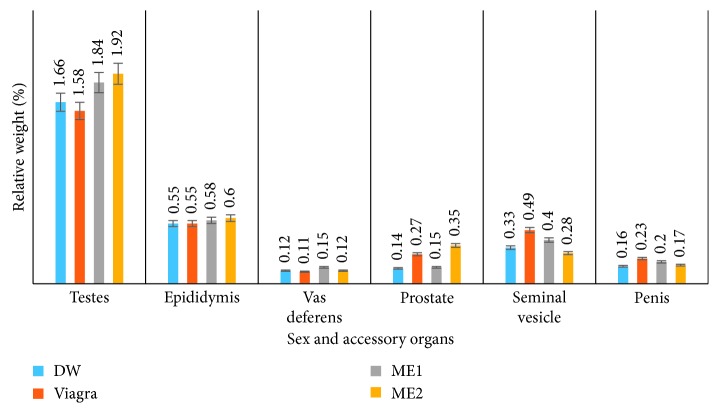
Effects of the leaf-methanolic extract of *P. arboreus* (ME1 and ME2) on the relative weight (%) of sex and accessory organs of amitriptyline-induced sexually impaired male rats. Values presented as mean ± SEM; DW: distilled water; ME1: methanolic extract dose 1 (46.5 mg/kg); ME2: methanolic extract dose 2 (93 mg/kg); Epidid: epididymis; (V) Def: vas deferens; S. Ves: seminal vesicles.

As summarized in Table 5 and illustrated in Figure 9, both doses (ME1 and ME2) of the leaf-methanolic extract induced an increase in relative weight of sex and accessory organs, with a significant (*p* < 0.05) effect on the testes compared to both Viagra and distilled water-treated rats. However, Viagra stimulated a nonsignificant (*p* < 0.05) increase in relative weight of the prostate, penis, and seminal vesicles, while ME2 provoked a significant (*p* < 0.05) increase in weight of prostate compared to ME1 and the distilled water groups.

#### 3.2.3. Effects on Plasma Concentrations of FSH, LH, and Testosterone

**Figure 10:**
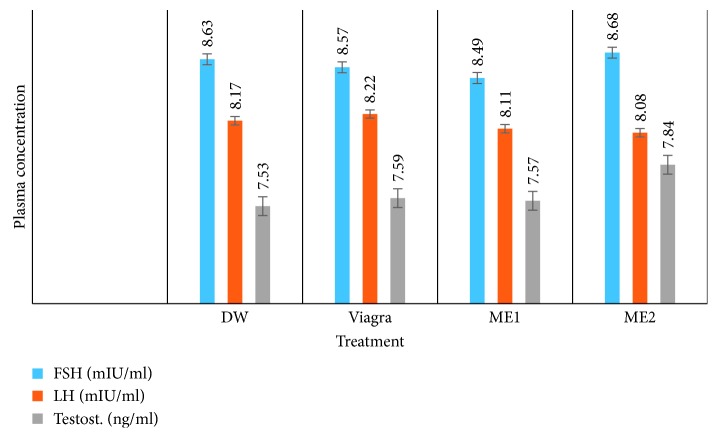
Effects of the leaf-methanolic extract of *P. arboreus* (ME1 and ME2) on plasma concentration of FSH, LH, and testosterone of amitriptyline-induced sexually impaired male rats. Values presented as mean ± SEM; DW: distilled water; ME1: methanolic extract dose 1 (46.5 mg/kg); ME2: methanolic extract dose 2 (93 mg/kg); FSH: follicle-stimulating hormone; LH: luteinizing hormone; Testost.: testosterone.

Oral treatment of the amitriptyline-induced sexually impaired male rats with either dose of the leaf-methanolic extract of *P. arboreus* had no significant effect on the plasma concentrations of the follicle-stimulating hormone (FSH), luteinizing hormone (LH), and testosterone (Table 6 and Figure 10).

#### 3.2.4. Acute Toxicity Test

No mortality and changes in the behavior were noticed in both the control and treated groups of rats right up to the dose of 5000 mg/kg. The food and water intake of the treated rats remained same as in the control animals.

## 4. Discussion

All categories of antidepressants have been implicated in the development of most forms of sexual dysfunction including decreased sexual interest (libido), erectile dysfunction (ED), impaired ejaculation, and impaired orgasm [4, 5]. In general, the tricyclic antidepressants (TCAs) and the monoamine oxidase inhibitors (MAOIs) seem to affect all stages of the male sexual function [26]. All antidepressants induce MSD by altering the availability and actions of neurotransmitters or hormones that intervene in the male sexual response cycle either at the central or the peripheral level. It is presumed that the more traditional tricyclic antidepressants (TCAs) may cause sexual dysfunction by altering the availability and actions of dopamine (DA) at the CNS [26]. Dopamine and testosterone are known to be implicated in sexual desire or interest or libido, stage 1 of the male sexual response cycle. The role of DA in mating behavior has been well studied in male rats, and data suggest that DA release in the medial preoptic area (MPOA) is essential for activation of adult male sexual behavior [27]. For masculine sex behavior in rats, it is critical that DA is not only present, but is released into the extracellular environment where it can bind to its postsynaptic receptors [27, 28]. As far as the regulation of copulatory behavior is concerned, testosterone has been associated with an increase in sexual behavior [29, 30]. Also, for normal sexual activity, testosterone is responsible for penile tumescence and rigidity as well as accessory muscles that help in improving penile rigidity and ejaculation [31]. Furthermore, according to [32] and [33], testosterone may enhance sexual behavior by increasing DA release in the MPOA of the hypothalamus and potentiating NO (nitric oxide) neurotransmission. Amitriptyline, a tricyclic antidepressant, upon chronic administration decreased libido (sexual interest), erectile function, and impaired ejaculation. This is translated by the significantly lower values of the sexual behavior parameters, relative weight of sex and accessory organs, and plasma hormonal concentrations recorded following prolonged treatment of sexually trained male rats with 10 mg/kg body weight of amitriptyline hydrochloride. These animals recorded increased time of hesitation towards receptive females and reduced number of mounts and intromissions. This decrease in sexual performance of sexually trained rats exposed to chronic administration of 10 mg/kg amitriptyline could be as a result of imbalance between dopamine secretion and availability.

Like in Viagra-treated animals, these parameters significantly improved in these animals upon treatment on day 1 and subsequent treatment with either dose of the leaf-methanolic extract of *P. arboreus,* compared to distilled water-treated animals. Significant decreases in ML and IL and significant increases in MF and IF were noted in animals treated with the plant extract at either dose, compared to the distilled water-treated group. MF and IF are regarded as indicators of libido or sexual desire, stage 1 of the male sexual cycle, as well as vigour and potency, while ML and IL are considered as indicators of sexual arousal, a stage influenced by dopamine and testosterone. The number of mounts (mount frequency, MF) reflects sexual motivation, whereas increase in the number of intromissions (intromission frequency, IF) shows the efficiency of erection, penile orientation, and the ease by which ejaculatory reflexes are activated [22]. The decreases in ML and IL as well as increases in MF and IF recorded in extract-treated rats throughout the treatment period indicate that libido and arousal were enhanced by both doses of the plant extract [34, 35]. We did not determine brain concentration of dopamine in the animals before and after treatment with different substances; in addition, testosterone bioassay revealed a non-significant effect in the plasma concentration level of testosterone in extract- and Viagra-treated animals, compared to the distilled water-treated rats. The leaf-methanolic extract of *P. arboreus* therefore likely improves sexual activity in amitriptyline-treated rats either through a dopamine-dependent mechanism or actions of substances mimicking testosterone, but not through increased testosterone secretion. Studies in laboratory animals such as rats have attributed the responsibility to many components of plant extracts as the possible bioactive agents increasing the endogenous testosterone level and enhancing male sexual behavior. Ginseng saponin has been shown to enhance libido and copulatory performance by acting directly on the CNS and gonadal tissues [36], and evidence indicates its potential to facilitate penile erection by directly inducing the vasodilation and relaxation of the penile corpora cavernosa via an NO-dependent mechanism [37], including arginase inhibition [38]. Amongst the phytoconstituents found in our plant extract are the saponins which have been reported to enhance sexual behavior in various animal models. The improvement in sexual function observed in this study might therefore be as a result of the presence of such compounds in the leaf-methanolic extract of *P. arboreus*. However, further studies are necessary to identify the active constituent (s) responsible for the sexual function improvement activities and the mechanisms in which these activities could be involved.

Rats treated with 46.5 mg/kg (ME1) dose of the extract recorded increased values of EL while those that received the 93 mg/kg (ME2) dose registered decreased values of EL; however, both doses induced a decrease in PEI values. Prolonged ejaculation is an indicator of prolonged coitus. PEI is regarded as an indicator of potency, libido, and potential to recover from exhaustion after the first ejaculatory series. These data clearly support the role of leaf-methanolic extract of *P. arboreus* in enhancing male sexual function and thus confirm its folk use. PL and MII (ICE) are important indices for evaluating the effect of drug administration on erectile function [39]. Both doses of the plant-leaf-methanolic extract induced an increase in PL and a decrease in MII (ICE) throughout the treatment period compared to the distilled water-treated group, though to a lesser degree than the Viagra-treated animals. This further shows that the leaf-methanolic extract of *P. arboreus* increases potency. The prolonged EL noticed with the 46.5 mg/kg dose and the increased penile erection (PL) noticed with both doses suggest the involvement of NO in the intervention [40].

Many activities of medicinal plants have been attributed to the phytocompounds found in these plants, and many bioactive components of plant extracts also exhibit aphrodisiac potentials by acting directly on the CNS to modulate the action of neurotransmitters and gonadal tissues in males or through vasodilation and the generation of NO, which can also change sexual behavior. In addition to saponins, the phytochemical screening of the leaf-methanolic extract of *P. arboreus* revealed the presence of alkaloids, flavonoids, steroids, phenols, and triterpenoids, a composition similar to most aphrodisiac plants, which have all been demonstrated to have sex-enhancing potentials. All these compounds have been reported to enhance male sexual performance through various mechanisms. Alkaloids are known to increase the dilation of blood vessels in the sexual organs [41]. Studies conducted by Ko et al. [42] indicate that flavonoids are phosphodiesterase (PDE) inhibitors. Phosphodiesterases are enzymes that break down cyclic AMP (cAMP), which activates synthesis of nitric oxide leading to vasodilatation thus increasing penile blood flow and sustaining penile erection [43]. Our plant extract probably acted through the intermediary of its phytoconstituents such as saponins to improve sexual performance in amitriptyline-induced sexually impaired male rats not through androgen secretion, but by acting directly on the CNS to modulate the action of DA and gonadal tissues or through vasodilation and the generation of NO, which changes sexual behavior.

The nonsignificant increase in plasma levels of testosterone in the extract-treated animals correlated with nonsignificant increase in FSH and LH plasma levels. Testosterone is synthesized and secreted by the Leydig cells of the testis under the influence of LH (luteinizing hormone, a gonadotrophin). Unfortunately, plasma levels of both LH and FSH were less significant, which means our extract had no effect on the Leydig cells. This further supports the fact that in this animal model, our extract does not act through androgen secretion.

The enhancements in the weights of sex and accessory organs of male rats are usually associated with androgenic activity and anabolic function. Androgens can stimulate the growth of accessory sexual organs (e.g., testis, seminal vesicles, and prostate) and increase their weights [44]. If certain drugs or natural compounds can increase the weights of accessory sexual organs, they are considered to possess androgenic properties [45]. Increase in relative weight of sex and accessory organs of animals treated with medicinal plants is often attributable to anabolic effects of raised testosterone levels, which cause an increase in metabolism, tissue generation, and muscle building. Testosterone has been reported to be useful for the histomorphometric development and maintenance of the testes and ultimately the biochemical process of sperm production [46]. Unfortunately, both extract-treated and control animals registered nonsignificant increase in plasma testosterone values. The significant increase in testicular weight noticed in ME2-treated rats could therefore result from the action of the phytoconstituents such as alkaloids, mimicking testosterone.

## 5. Conclusion

Leaf-methanolic extract of *P. arboreus* improves sexual function in amitriptyline-induced sexually impaired male rats and is nontoxic. Its mechanism of action does not involve androgen secretion, but could be through testosterone-mimicking action, NO-dependent or directly on the CNS. It could constitute a potential solution to AIMSD.

## Figures and Tables

**Table 1:** Effects of the leaf-methanolic extract of *P. arboreus* (ME1 and ME2) on mount latency (ML) (s) and intromission latency (IL) (s) of amitriptyline-induced sexually impaired male rats.

		Treatment			
Parameter	Day	DW	Viagra	ME1	ME2
ML (s)	1	174.60 ± 40.15^ae^	116.60 ± 25.73^be^	149.20 ± 30.88^ce^	125.40 ± 25.18^de^
	7	158.60 ± 40.80^af^	83.71 ± 20.22^bf^	93.40 ± 30.53^cf^	90.00 ± 20.43^cf^
	14	103.80 ± 50.52^ag^	71.00 ± 20.66^bg^	82.55 ± 35.03^bg^	78.23 ± 25.98^bg^
	21	87.40 ± 43.62^ah^	69.60 ± 22.38^bg^	77.40 ± 35.30^bg^	75.18 ± 27.86^bg^

IL (s)	1	192.66 ± 45.83^ae^	128.40 ± 22.68^be^	163.80 ± 40.20^ce^	134.17 ± 27.56^de^
	7	177.34 ± 45.50^ae^	92.00 ± 20.47^bf^	89.40 ± 37.11^bf^	85.22 ± 23.69^bf^
	14	113.67 ± 50.38^af^	85.60 ± 23.93^bf^	94.51 ± 39.23^cf^	88.20 ± 24.42^bf^
	21	102.11 ± 52.11^af^	77.20 ± 25.88^bf^	87.63 ± 35.76^bf^	85.60 ± 23.93^bf^

Values presented as mean ± SEM; DW: distilled water; ME1: methanolic extract dose 1 (46.5 mg/kg); ME2: methanolic extract dose 2 (93 mg/kg); s: seconds; on the same row, values with same letters (a–d) are not significantly different; on the same row, values with different letters are significantly different; on the same column, values with the same letters (e–h) are not significantly different; on the same column, values with different letters are significantly different; ML: mount latency (ML); IL: intromission latency.

**Table 2:** Effects of the leaf-methanolic extract of *P. arboreus* (ME1 and ME2) on mount frequency (MF) and intromission frequency (IF) of amitriptyline-induced sexually impaired male rats.

	Treatment				
Parameter	Day	DW	Viagra	ME1	ME2
MF	1	14.60 ± 1.20^ae^	18.29 ± 2.50^ae^	16.07 ± 1.96^ae^	16.33 ± 2.36^ae^
	7	17.00 ± 1.00^ae^	20.00 ± 2.30^ae^	17.80 ± 1.80^ae^	17.31 ± 2.50^ae^
	14	17.80 ± 1.70^ae^	22.20 ± 2.50^ae^	20.00 ± 2.00^ae^	21.00 ± 2.30^ae^
	21	18.00 ± 1.80^ae^	23.60 ± 1.90^ae^	18.51 ± 1.50^ae^	16.60 ± 1.00^ae^

IF	1	11.40 ± 1.60^ae^	17.60 ± 1.00^be^	15.00 ± 1.00^ae^	13.80 ± 4.00^ae^
	7	13.40 ± 1.00^ae^	19.21 ± 1.00^abe^	14.20 ± 1.20^ae^	24.20 ± 4.00^bf^
	14	14.20 ± 1.20^ae^	18.20 ± 1.00^ae^	17.40 ± 1.20^ae^	18.00 ± 4.00^ae^
	21	15.36 ± 0.70^ae^	20.60 ± 1.00^ae^	15.80 ± 1.60^ae^	18.20 ± 4.40^ae^

Values presented as mean ± SEM; DW: distilled water; ME1: methanolic extract dose 1 (46.5 mg/kg); ME2: methanolic extract dose 2 (93 mg/kg); on the same row, values with same letters (a-b) are not significantly different; on the same row, values with different letters are significantly different; on the same column, values with the same letters (e-f) are not significantly different; on the same column, values with different letters are significantly different; MF: mount frequency; IF: intromission frequency.

**Table 3:** Effects of the leaf-methanolic extract of *P. arboreus* (ME1 and ME2) on ejaculation latency (EL) (s) and postejaculatory interval (PEI) (s) of amitriptyline-induced sexually impaired male rats.

	Treatment				
Parameter	Day	DW	Viagra	ME1	ME2
EL (s)	1	808.00 ± 120.78^ae^	753.80 ± 12.13^be^	648.40 ± 165.76^ce^	755.80 ± 105.95^be^
	7	648.40 ± 98.76^af^	726.00 ± 25.64^be^	735.20 ± 170.71^bf^	491.60 ± 120.38^cf^
	14	692.20 ± 110.30^af^	735.20 ± 15.71^be^	422.40 ± 171.83^cg^	649.20 ± 106.83^ag^
	21	858.20 ± 91.45^ae^	703.40 ± 25.59^be^	798.60 ± 180.02^ah^	692.20 ± 120.30^cg^

PEI (s)	1	421.20 ± 65.01^ae^	369.00 ± 30.16^be^	477.00 ± 20.99^ce^	493.20 ± 80.98^ce^
	7	477.80 ± 55.99^af^	292.40 ± 28.00^be^	443.40 ± 20.17^ae^	443. 20 ± 75. 26^ae^
	14	537.80 ± 70.51^ag^	332.00 ± 35.59^be^	413.40 ± 25.39^ce^	359.60 ± 63.79^bf^
	21	417.40 ± 60.38^ae^	351.60 ± 30.02^be^	437.40 ± 29.38^ae^	332.00 ± 75.59^bf^

Values presented as mean ± SEM; DW: distilled water; ME1: methanolic extract dose 1 (46.5 mg/kg); ME2: methanolic extract dose 2 (93 mg/kg); s: seconds; on the same row, values with same letters (a–c) are not significantly different; on the same row, values with different letters are significantly different; on the same column, values with the same letters (e–g) are not significantly different; on the same column, values with different letters are significantly different; EL: ejaculation latency (s); PEI: postejaculatory interval (s).

**Table 4:** Effects of the leaf-methanolic extract of *P. arboreus* (ME1 and ME2) on penile licking (PL) and mean intromission interval (MII) (s) of amitriptyline-induced sexually impaired male rats.

	Treatment				
Parameter	Day	DW	Viagra	ME1	ME2
PL	1	3.20 ± 0.40^ae^	5.21 ± 1.00^ae^	3.60 ± 0.50^ae^	4.40 ± 0.80^ae^
	7	3.60 ± 0.40^ae^	6.83 ± 1.00^be^	3.80 ± 0.40^ae^	4.92 ± 0.80^ae^
	14	3.80 ± 0.40^ae^	7.17 ± 1.00^ae^	4.20 ± 0.40^ae^	5.75 ± 0.75^ae^
	21	3.72 ± 0.20^ae^	6.20 ± 0.90^be^	4.42 ± 0.28^ae^	5.93 ± 0.90^ae^

MII (s)	1	71.60 ± 15.50^ae^	32.80 ± 8.00^be^	43.60 ± 4.00^ce^	54.80 ± 15.00^de^
	7	42.20 ± 16.46^af^	51.00 ± 10.80^bf^	53.40 ± 6.15^bf^	20.00 ± 14.32^cf^
	14	42.60 ± 15.91^af^	38.00 ± 8.36^be^	53.80 ± 5.10^cf^	24.40 ± 4.97^df^
	21	61.00 ± 14.72^ag^	38.60 ± 7.45^be^	51.80 ± 6.11^cf^	38.00 ± 16.36^bg^

Values presented as mean ± SEM; DW: distilled water; ME1: methanolic extract dose 1 (46.5 mg/kg); ME2: methanolic extract dose 2 (93 mg/kg); s: seconds; on the same row, values with same letter (a–d) are not significantly different; on the same row, values with different letters are significantly different; on the same column, values with the same letters (e-f) are not significantly different; on the same column, values with different letters are significantly different; PL: penile licking; MII: mean intromission interval (s).

**Table 5:** Effects of the methanolic extract (ME1 and ME2) of *P. arboreus* on the relative weight of sex and accessory organs of amitriptyline-induced sexually impaired male rats.

Treatment	Testes	Epididymis	Vas deferens	Prostate	Seminal vesicle	Penis
D.W. (10 ml/kg)	1.66 ± 0.12^a^	0.55 ± 0.04^a^	0.12 ± 0.01^a^	0.14 ± 0.05^a^	0.33 ± 0.05^a^	0.16 ± 0.02^a^
Viagra	1.58 ± 0.14^a^	0.55 ± 0.06^a^	0.11 ± 0.04^a^	0.27 ± 0.05^a^	0.49 ± 0.1^a^	0.23 ± 0.04^a^
ME1	1.84 ± 0.22^b^	0.58 ± 0.05^a^	0.15 ± 0.03^a^	0.15 ± 0.03^a^	0.40 ± 0.1^a^	0.20 ± 0.02^a^
ME2	1.92 ± 0.20^b^	0.60 ± 0.04^a^	0.12 ± 0.01^a^	0.35 ± 0.06^b^	0.28 ± 0.05^a^	0.17 ± 0.01^a^

Values presented as mean ± SEM; DW: distilled water; ME1: methanolic extract dose 1 (46.5 mg/kg); ME2: methanolic extract dose 2 (93 mg/kg); s: seconds; on the same row, values with same letters (a-b) are not significantly different; on the same row, values with different letters are significantly different.

**Table 6:** Effects of the leaf-methanolic extract of *P. arboreus* (ME1 and ME2) on the plasma concentrations of FSH, LH, and testosterone of amitriptyline-induced sexually impaired male rats.

Hormone	Treatment			
	DW	Viagra	ME1	ME2
FSH (mIU/ml)	8.63 ± 0.11	8.57 ± 0.04	8.49 ± 0.08	8.68 ± 0.07
LH (mIU/ml)	8.17 ± 0.05	8.22 ± 0.09	8.11 ± 0.09	8.08 ± 0.64
Testosterone (ng/ml)	7.53 ± 0.12	7.59 ± 0.14	7.57 ± 0.11	7.84 ± 0.22

Values presented as mean ± SEM; DW: distilled water; ME1: leaf-methanolic extract dose 1 (46.5 mg/kg); ME2: leaf-methanolic extract dose 2 (93 mg/kg); FSH: follicle-stimulating hormone; LH: luteinizing hormone.

## Data Availability

The data used to support the findings of this study are included within the article.
